# Risk Factors for the Recurrence of Massive Hemoptysis Treated With Bronchial Artery Embolization: A Retrospective Study

**DOI:** 10.1111/crj.70187

**Published:** 2026-04-27

**Authors:** Tianhua Yue, Ling Li, Zhengyu Yue

**Affiliations:** ^1^ Department of Interventional Radiology Yancheng First Hospital Affiliated of Nanjing University Medical College Yancheng Jiangsu China; ^2^ Jiangsu Medical College Yancheng Jiangsu China; ^3^ Department of Interventional Radiology, Jianhu County People's Hospital Jianhu Jiangsu China; ^4^ First School of Clinical Medicine Shandong Second Medical University Weifang Shandong China

**Keywords:** bronchial artery embolization, CT angiography, massive hemoptysis, recurrence

## Abstract

**Background:**

Bronchial artery embolization (BAE) is preferred for massive hemoptysis. However, the suboptimal short‐term and long‐term therapeutic outcomes have necessitated the initiation of this study. The aim of this study was to identify risk factors that influence the recurrence of massive hemoptysis after BAE and determine active prevention and control measures to reduce recurrence.

**Methods:**

Between January 2019 and November 2024, a total of 162 patients with massive hemoptysis underwent BAE, of whom 47 required re‐embolization due to recurrence. Baseline data of patients, technical success, clinical success, recurrence, complications, and other relevant information were collected from outpatient and inpatient medical records and subsequently analyzed. The Cox regression analysis and Forest map were employed to analyze the risk factors associated with recurrence of massive hemoptysis after BAE.

**Results:**

Findings suggested that the technical success rate was (160/162) 98.76% and clinical success rate was (115/162) 70.99% during the 12‐month follow‐up. Mean recurrence‐free time was 26 ± 3.43 days (95% CI: 19.28–32.72) among 47 patients who experienced recurrence following BAE. Multivariate Cox regression analysis showed that the risk factors for early recurrence of hemoptysis following BAE were the extent of destroyed lung (OR = 0.562 [95% CI: 0.325–0.973], *p* = 0.04), whether preoperative computed tomography angiography (CTA) (OR = 0.204 [95% CI: 0.083–0.499], *p* = 0.001), or technical factors (OR = 4.621 [95% CI: 1.936–11.028], *p* = 0.001), while the risk factor for late recurrence was the progression of underlying diseases (OR = 6.071 [95% CI: 1.968–18.731], *p* = 0.002). However, the overall risk factors for recurrent hemoptysis after BAE included the extent of destroyed lung (OR = 0.606 [95% CI: 0.404–0.91], *p* = 0.016), whether preoperative CTA (OR = 0.49 [95% CI: 0.266–0.905], *p* = 0.023), technical factors (OR = 2.176 [95% CI: 1.089–4.348], *p* = 0.028), and the progression of underlying diseases (OR = 1.958 [95% CI: 1.047–3.662], *p* = 0.035. There were no major complications related to BAE requiring immediate treatment, and only minor complications were observed.

**Conclusion:**

This study preliminarily concludes that the extent of destroyed lung, whether preoperative CTA, technical factors, and the progression of underlying diseases are independent risk factors associated with hemoptysis recurrence after BAE. Through comprehensive preoperative assessments, individualized embolization strategies, and proactive postoperative management of underlying diseases, the risk of recurrent hemoptysis can be significantly reduced.

## Graphical abstract

This study preliminarily concludes that the extent of destroyed lung, whether preoperative CTA, technical factors, and the progression of underlying diseases are independent risk factors associated with hemoptysis recurrence after bronchial artery embolization (BAE). Through comprehensive preoperative assessments, individualized embolization strategies, and proactive postoperative management of underlying diseases, the risk of recurrent hemoptysis can be significantly reduced.

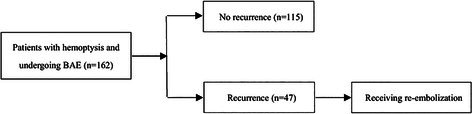

**Abbreviations**
BAE: bronchial artery embolization
COPD: chronic obstructive pulmonary disease
CTA: computed tomography angiography
NBCA: *N*‐butyl‐2‐cyanoacrylate
PVA: polyvinyl alcohol

## 1 Introduction

Hemoptysis is defined as the expectoration of blood originating from tracheobronchial or pulmonary parenchyma, alone or in combination with mucus. Hemoptysis is encountered as a clinical symptom, which can be induced by various underlying diseases. Etiological surveys have shown that the most common causes of hemoptysis are tuberculosis, bronchiectasis, chronic obstructive pulmonary disease (COPD), fungal infections, pneumonia, malignancy, cystic fibrosis, cryptogenic hemoptysis, and even pulmonary arterial pseudoaneurysm, all of which vary depending on geographic region [1, 2, 3]. It has been found that inflammation, hypoxia, and neoplasia can result in recruitment of systemic blood supply, neovascularization, and abnormal vasculature formation via secretion of proangiogenic factors such as vascular endothelial growth factor and angiopoietin‐1 [4, 5]. Furthermore, repeated inflammation can lead to bronchial artery hypertrophy, distortion, aneurysm formation, and systemic circulation–pulmonary vascular anastomosis or arterial fistula formation. These vessels are usually fragile and prone to rupture into the airways, resulting in hemoptysis. Generally, hemoptysis can be classified as mild, moderate, and massive. There is no consensus on the definition of massive hemoptysis, but it is generally considered that coughing more than 100 mL of blood once or more than 500 mL within 24 h is massive hemoptysis [6]. Patients with massive hemoptysis are at high risk for respiratory failure because massive bleeding may obstruct the airways [7, 8]. Jin et al. [9] reported that the mortality rate of massive hemoptysis was about 6.5%–38%, with the cause of death usually from asphyxiation, not exsanguination.

Massive hemoptysis is a life‐threatening respiratory emergency that requires immediate intervention to save lives. Mild and moderate hemoptysis can be controlled by medical treatment, whereas poor response to vasoactive drugs alone for massive hemoptysis requires more aggressive management, such as bronchoscopy, surgery, or even bronchial artery embolization (BAE). Bronchoscopy has clinical significance in the diagnosis and treatment of massive hemoptysis. However, the hemostatic effect of bronchoscopy is limited due to the limited suction capacity, blurred visual field, and the bleeding site beyond the reach of bronchoscopy. Surgery was once considered the definitive cure for hemoptysis; however, the mortality rate of patients who received urgent surgery was approximately 40% higher compared to the elective procedures [10]. Given the relatively high surgical risk factors—such as advanced age, impaired lung function, bilateral lung lesions, and other comorbidities—BAE is a better option. In some cases, BAE may be considered an assistant therapy for massive hemoptysis, which provides more time for other treatment options during an emergency.

The lungs are perfused with dual blood supplies: bronchial artery and pulmonary artery. Considering that in nearly 90% of cases, the culprit vessel of hemoptysis is the bronchial artery, BAE has become the preferred treatment for massive hemoptysis [11]. BAE is a minimally invasive endovascular procedure with low morbidity and mortality, high technical success and clinical success, but a high recurrence rate is its main shortage, which requires repeat embolization or surgery. Tao et al. [12] investigated a systematic review and meta‐analysis of 32 articles and found that the recurrence rates ranged from 8.0% to 55.1%. Abid et al. [13] reported that the recurrence rate of hemoptysis ranged from 1% to 27% within 1 month and 10% to 55% between 1 and 46 months after BAE. At our centers, BAE has been routinely employed for the management of massive hemoptysis over many years; however, the recurrence rate is in line with the above studies. In this retrospective analysis, we seek to identify the risk factors that influence the recurrence of massive hemoptysis after BAE and determine active prevention and control measures to reduce recurrence.

## 2 Materials and Methods

### 2.1 Patient Selection

**FIGURE 1:**
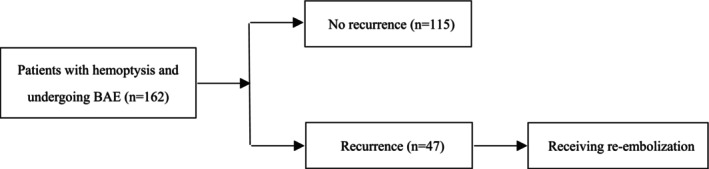
Flow chart of patients inclusion. Abbreviation: BAE, bronchial artery embolization.

Between January 2019 and November 2024, 162 patients with massive hemoptysis who underwent BAE at Yancheng First Hospital Affiliated of Nanjing University Medical College and Jianhu County People's Hospital, were enrolled for this study. The indications and contraindications for BAE were determined through previous medical history, physical examination, laboratory studies, and imaging. The inclusion criteria were adult patients who presented with massive hemoptysis (hemoptysis volume >100 mL in one episode or hemoptysis volume >500 mL within 24 h) and follow‐up period continued for 1 year. The exclusion criteria were hemoptysis caused by pulmonary circulation or nonpulmonary promoting factors (i.e., coagulation dysfunction, thrombocytopenia). This retrospective study was approved by the ethics committee of Jianhu County People's Hospital according to the standards of Declaration of Helsinki (Ethical Approval JY‐LL‐202401‐K023). Yancheng First Hospital Affiliated of Nanjing University Medical College was informed of the study. Due to the retrospective nature of the study, the ethics committee of Jianhu County People's Hospital waived the need for obtaining informed consent for clinical research. However, all patients included in this study provided written informed consent prior to undergoing BAE. The data of all patients obtained depending upon our hospital files were anonymized and deidentified prior to analysis in this study. Figure 1 showed the flow chart of patients' inclusion.

### 2.2 Procedure

A meticulous pre‐procedure comprehensive evaluation of symptoms, etiology, and extent of lung involvement was performed in all patients. Whenever feasible, CT angiography (CTA) should be conducted preoperatively to identify the culprit vessels. All procedures were performed via a common femoral artery approach, under local anesthesia, percutaneously, using the Seldinger technique. The 5‐French (F) vascular sheath was inserted through femoral artery puncture and various types of angiography catheters (i.e., Cobra‐2, Simmons‐1, or Mikaelsson) were introduced due to the diversity of culprit vessels. Selective catheterization and hand‐injection angiography were then performed to further identify the culprit vessels. The visualized culprit vessels demonstrated active extravasation of the contrast agent or abnormal morphology (i.e., hypertrophied, irregular and tortuous bronchial arteries, focal hyperemia and hypervascularity, arterio‐venous shunts, bronchial artery aneurysms) and received embolization. If the angiography catheter was not stable, or if we were unsure of the anastomosis to anterior spinal artery, a 2.9‐F coaxial microcatheter was introduced and advanced through the culprit arteries at the most possible distal point to avoid embolization of the important side branches. The position was confirmed by repeated angiogram with microcatheter and embolic materials were injected. The embolic materials were injected gradually in a pulsatile manner with small‐volume syringes under fluoroscopic real‐time imaging to prevent reflux. The end point for embolization was that the contrast column had sustained at least five heartbeats and the presence of blood stasis. The 5‐F vascular sheath was removed postprocedure, and manual compression was performed for hemostasis. All these patients were then monitored and observed for a period of 24 h postprocedure, including hemodynamic support, airway protection, and neurological evaluation.

### 2.3 Evaluation Criteria

All the patients were followed up for 1 year postprocedure, if feasible. The electronic medical records of each patient by documented clinic and hospital visits were analyzed to collect data on relevant aspects. The outcomes of the procedure were assessed, which included technical success, clinical success, recurrence, additional treatments, and major complications. The technical success was defined as the cessation and exclusion of the bleeding focus from the targeted vessel as confirmed by angiography. The clinical success was defined as either complete cessation or reduction of hemoptysis to minimal levels (e.g., sputum with trace blood staining), resulting in clinical improvement, with no further intervention required beyond standard medical management after embolization. Recurrence was defined as significant hemoptysis during the follow‐up period, requiring urgent medical intervention such as repeat BAE or surgery. Major complications involving vital or functional patient prognosis were defined as sequelae, which were permanent, even death, requiring further treatment. Nevertheless, minor complications were self‐limited and did not usually require treatment.

### 2.4 Statistical Analysis

The statistical software package SPSS 24 was used for statistical analysis. Continuous variables were expressed as means ± standard deviation (mean ± SD) according to normal distribution, while categorical variables were displayed using numbers and percentages (*n*, (%)). The significance of differences was analyzed using the *t*‐test to compare continuous variables based on normal distribution, while the chi‐square test was used to evaluate categorical variables. The cumulative recurrence rate after BAE was assessed using the Kaplan–Meier method and Cox regression analysis was conducted on the risk factors associated with hemoptysis recurrence after BAE. A Forest map was created using GraphPad Prism version 9.5 software. *P* < 0.05 was considered statistically different.

## 3 Results

### 3.1 Angiographic Findings Following the Responsible Vessels

All patients underwent BAE involving 432 culprit vessels, including orthotopic and heterotopic bronchial arteries, or nonbronchial systemic collaterals. The origin of heterotopic bronchial arteries or nonbronchial systemic collaterals included the subclavian arteries (*n* = 22), thyrocervical trunk (*n* = 13), aortic arch (*n* = 2), intercostal arteries (*n* = 45), internal mammary arteries (*n* = 23), phrenic artery (*n* = 12), and left gastric artery (*n* = 5).

### 3.2 The Therapeutic Efficacy by BAE

**FIGURE 2:**
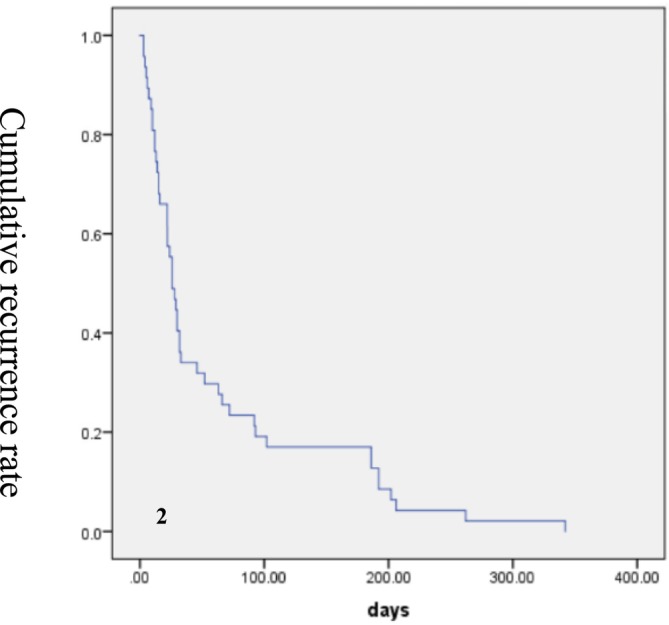
The Kaplan–Meier curve of cumulative recurrence rate among 47 patients who experienced recurrence following BAE.

**FIGURE 3:**
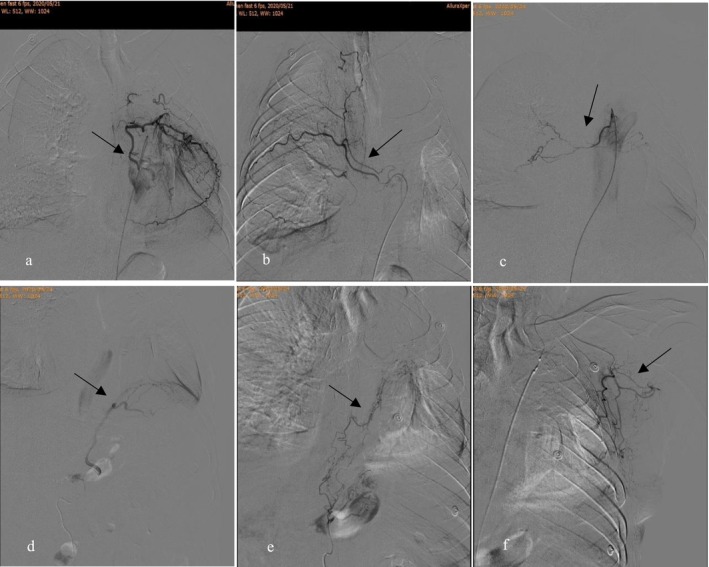
A 74‐year‐old man with history of bronchiectasis presenting with recurrent massive hemoptysis after BAE. (a) Selective intercostal angiogram (digital subtraction angiographic image) prior to first embolization showing tortuous and hypervascular intercostal artery (arrow). (b) Right bronchial angiogram showing the presence of a right intercostobronchial trunk (arrow) from which arises an intercostal artery and a bronchial artery. (c) Selective bronchial angiogram prior to repeat embolization showing multiple small tortuous branches supplying a hypervascular lesion (arrow). (d) Selective left phrenic angiogram showing small tortuous branches supplying the same hypervascular lesion (arrow). (e) Selective left gastric artery angiogram showing tortuous branches in the same patient (arrow). (f) Selective left subclavian artery angiogram showing multiple small tortuous branches (arrow).

**FIGURE 4:**
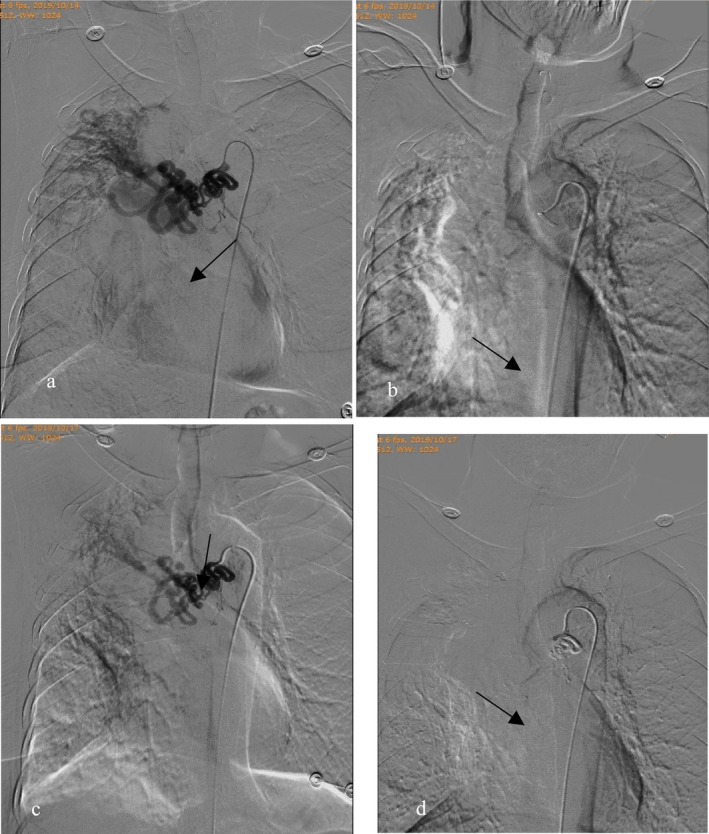
A 74‐year‐old woman with history of bronchiectasis presenting with recurrent massive hemoptysis after BAE. (a) Selective right bronchial angiogram (digital subtraction angiographic image) prior to first embolization showing a dilated, tortuous, and hypervascular vessel with hyperemia (arrow). (b) Selective angiogram of the right bronchial artery postembolization with the use of PVA and gelfoam shows pruning of the vasculature (arrow). (c) Repeat right bronchial angiogram prior to second embolization still showing hypertrophied and tortuous right bronchial artery giving a corkscrew appearance (arrows). (d) Right bronchial angiogram done postembolization with the use of NBCA showing vascular stasis along with disappearance of the hypervascular lesion (arrow).

Technical success was obtained in 160 of 162 (98.76%) patients. The technical failure was not a complete failure, but a partial embolization failure after embolization of most target vessels. In the two instances of technical failure, the reason was attributed to excessive narrowness of the target vessels, which prevented successful catheter insertion. Then, these two patients subsequently improved after intravenous hemostatic agents. During the follow‐up period, clinical success was attained in 115/162 (70.99%) of patients. Figure 2 presented the Kaplan–Meier curve, which illustrated the cumulative recurrence rate among 47 patients who experienced recurrence following BAE and mean recurrence‐free time was 26 ± 3.43 days (95% CI: 19.28–32.72). Then, these recurrent patients received re‐embolization, following which hemoptysis was successfully controlled (Figures 3 and 4). Multivariate Cox regression analysis showed that the risk factors for early recurrence of hemoptysis (≤1 month) following BAE were the extent of destroyed lung, whether preoperative CTA, and technical factors, while the risk factor for late recurrence (>1 month) was the progression of underlying diseases (Table 1).

**TABLE 1:** Multivariate Cox regression analyses of risk factors associated with early recurrence and late recurrence.

Factors	Early recurrence (≤1 month)			Late recurrence (>1 month)		
	HR	95% CI	*p*	HR	95% CI	*p*
Age	0.985	0.949–1.024	0.449	1.050	0.999–1.102	0.053
Gender	1.003	0.449–2.242	0.994	1.161	0.403–3.342	0.783
BMI	1.302	0.520–3.264	0.573	1.049	0.324–3.394	0.936
Hypertension	1.320	0.527–3.307	0.554	1.358	0.447–4.128	0.590
Diabetes	0.872	0.333–2.286	0.781	1.905	0.600–6.050	0.274
Smoking history	1.259	0.513–3.089	0.615	0.842	0.317–2.235	0.730
Etiology of hemoptysis	0.717	0.495–1.039	0.079	0.874	0.588–1.299	0.505
Destroyed lung	0.562	0.325–0.973	0.040	0.602	0.304–1.194	0.146
Preoperative CTA	0.204	0.083–0.499	0.001	2.295	0.732–7.196	0.154
Technical factors	4.621	1.936–11.028	0.001	0.175	0.021–1.455	0.107
Progression of underlying diseases	1.287	0.551–3.007	0.561	6.071	1.968–18.731	0.002

Abbreviations: BMI, body mass index; CTA, CT angiography.

**FIGURE 5:**
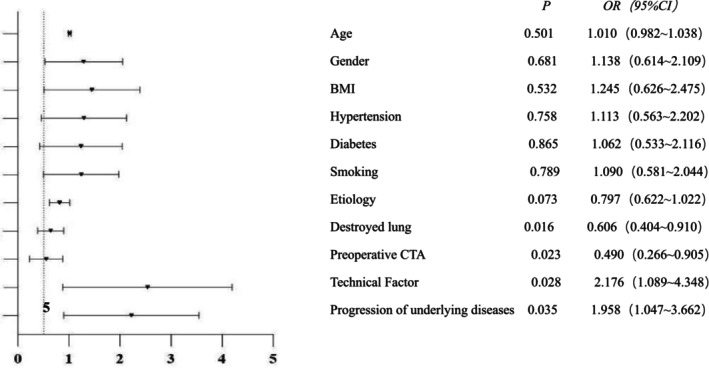
Multivariate Cox regression analyses of risk factors associated with recurrent hemoptysis.

Then, all patients were divided into the observation group (Recurrence) and the control group (No recurrence) based on whether there was recurrent hemoptysis. The clinical characteristics of all patients were listed in Table 2. The selected 11 variables were used for univariate analysis and the results showed that there were statistically significant differences in the extent of destroyed lung, whether preoperative CTA, technical factors, and the progression of underlying diseases between both groups (*p* < 0.05). Multivariate Cox regression analysis further demonstrated that the extent of destroyed lung, whether preoperative CTA, technical factors, and the progression of underlying diseases were independent risk factors for hemoptysis recurrence (Figure 5).

**TABLE 2:** Clinical characteristics of patients.

Factors	Observation group (*n* = 47)	Control group (*n* = 115)	*p*
Age (years)	67.49 (11.78)	67.60 (11.86)	0.98
Gender, *n* (%)			0.61
Male	29	66	
Female	18	49	
BMI, *n* (%)			0.81
<24 kg/m^2^	32	76	
≥24 kg/m^2^	15	39	
Hypertension, *n* (%)			0.58
Yes	30	68	
No	17	47	
Diabetes, *n* (%)			0.72
Yes	28	65	
No	19	50	
Smoking history, n (%)			0.93
Yes	25	62	
No	22	53	
Etiology of hemoptysis, *n* (%)			0.85
Bronchiectasis	15	37	
Tuberculosis	11	34	
Pneumonia	9	23	
Malignancy	7	12	
Fungal infection	5	9	
Destroyed lung, *n* (%)			0.006
Mild	10	42	
Moderate	10	38	
Severe	27	35	
Preoperative CTA, *n* (%)			0.016
Yes	21	75	
No	26	40	
Technical factors, *n* (%)			0.021
Yes	21	30	
No	26	85	
Progression of underlying diseases, *n* (%)			0.007
Yes	25	35	
No	22	80	

*Note:* Data were presented as mean ± SD and *n* (%).

Abbreviations: BMI, body mass index; CTA, CT angiography; SD, standard deviation.

### 3.3 Complications

There were no major complications related to embolization requiring immediate treatment, and only minor complications were observed. The most common symptoms were transient chest/back pain with mild fever (*n* = 26, 16.05%), which resolved spontaneously or upon administration of oral analgesic–antipyretic. Transient dysphagia was observed (*n* = 6, 3.7%), which was self‐limited. No cases of transverse myelitis had been reported. Neurological complications in the form of bilateral lower limb paraplegia and paresthesia due to anterior cord ischemia did not occur. Access site complications were seen (*n* = 3, 1.85%) in the form of hematoma formation, which had resolved by manual compression. Two patients presented urticaria due to allergy to the contrast media. No contrast‐induced nephropathy was observed. There were no BAE‐related deaths in this study.

## 4 Discussion

Through univariate and multivariate analyses, this study identified the extent of destroyed lung, whether preoperative CTA, technical factors, and the progression of underlying diseases as independent risk factors associated with hemoptysis recurrence after BAE. Patients with destroyed lung are prone to hemoptysis. Small amounts of hemoptysis are often self‐limiting or can be controlled with medication, but the severely destroyed lung can lead to massive hemoptysis. The aggravation of destroyed lung can result from persistent erosion of lung tissue by tuberculosis bacteria or cancer, poor control over lung infections. These are identified as the main reasons for hemoptysis recurrence. Zhao et al. [14] had proposed that the mechanisms by which destroyed lung led to massive hemoptysis included promotion of bronchodilation, invasion of adjacent blood vessels, and rupture of aneurysms. Wang et al. [15] reported that the proper esophageal artery, inferior phrenic artery, and intercostal artery were susceptible to systemic–pulmonary artery shunt when subjected to inflammatory stimulation in patients with destroyed lung. Figure 3 of this study demonstrated that repeat angiography identified the inferior phrenic artery, left gastric artery, and subclavian artery as culprit vessels to recurrent hemoptysis. This finding underscores the importance of thoroughly identifying all potential culprit vessels in clinical evaluation.

Bronchial arteries are the main vessels responsible for hemoptysis, but heterotopic bronchial artery or nonbronchial systemic collaterals are not infrequent findings in diseased lung, which also need embolization to achieve a good outcome [16]. All the culprit vessels of massive hemoptysis have always been a concern to interventionists when doing a pre‐procedural evaluation, which can be done via CTA. CTA could not only identify the extent and underlying cause of pulmonary disease but also visualize the vessel anatomy and determine the culprit vessels. CTA can simultaneously be considered a roadmap for BAE, possibly explaining the high technical success rate and reducing the procedural and fluoroscopic time. Zhang et al. [17] suggested that CTA should be recommended prior to BAE, as it showed the diagnostic value for detecting culprit vessels. However, preoperative CTA cannot be performed because of the limited time in massive hemoptysis, which requires understanding the anatomical location of bronchial arteries and following the proximity principle. Most (approximately 70%) bronchial arteries arise from the descending thoracic aorta, commonly between the T5 and the T6 vertebral plane, 1–2 cm above or below the carinal level and may be multiple on one side or the other. When not assisted by CTA, bronchial arteries are usually sought and angiographically examined between the levels of the T5–T6 vertebrae. If there is difficulty in finding the bronchial artery, a thoracic aortography can be performed to localize them. Except for bronchial arteries, examination of other systemic arteries that may be the source of bleeding on the suspected side should be considered. Dorji et al. [18] reported that for upper lobe disease, subclavian artery angiography should be done to identify possible coexistent nonbronchial systemic collaterals, especially in recurrent hemoptysis. Likewise, the inferior phrenic artery was also evaluated for lower lobe disease. If no culprit arteries are found, pulmonary angiography should be considered. Yang et al. [19] advocated dual‐vessel intervention including bronchial or pulmonary arterial embolization to manage massive hemoptysis caused by cavitary lung lesions. In this study, except for 66 patients who were taken directly for embolization because of unstable hemodynamics, all the other patients received preoperative CTA. Among these 66 patients, 26 cases experienced hemoptysis again.

Technical factors as another risk factor for hemoptysis recurrence after BAE includes incomplete embolization, recanalization of the embolized vessel and the opening of collateral branches, involving embolization methods and embolic materials [20]. Dessajan et al. [21] believed that the improvement in technical factors reflected improved operator expertise and advances in super‐selective catheterization and embolization techniques. In this study, certain patients experiencing recurrence did not undergo microcatheter‐guided superselective catheterization followed by stepwise multiple embolization, leading to incomplete embolization and subsequent recruitment of collateral circulation, which may have contributed to recurrence. Among the recurrent cases, CTA or descending thoracic aortogram using a 5‐F pig tail catheter using nonionic contrast medium was performed prior to re‐embolization to identify culprit vessels. Following selective catheterization of the culprit vessels, angiograms were performed to confirm their anatomy, and special attention should be paid to looking for the anterior spinal artery, which has a characteristic hairpin appearance and can arise from the common intercostal bronchial trunk. Then, culprit vessels were superselectively catheterized with a 2.9‐F microcatheter and a gradual multiple embolization was performed from the parenchymal bed of abnormality initially to higher order or even the open collateral branches with various embolization materials. Blood stasis or reflux of injected embolic materials indicated the endpoint for embolization.

The study by Tayal et al. [22] indicated that in addition to superselective catheterization with microcatheter, a gradual multiple embolization with decremental or incremental embolization materials size was helpful to control massive hemoptysis. Li et al. [23] reported that the embolic material used for BAE was a factor attributing to the recurrence of hemoptysis. Although there is no consensus on the optimal embolic materials for BAE, various embolic materials can be used to occlude the hemoptysis‐associated arteries, each with its own advantages and disadvantages. Gelfoam is the most conventional embolization material, which may be attributed to its low cost, wide availability, and ease of use during emergencies. Nagano et al. [24] reported that BAE using gelfoam had short‐ and long‐term hemostatic efficacy for treating cryptogenic hemoptysis without any severe complications. However, gelfoam is relatively transient and gets reabsorbed in human tissues in 2–6 weeks, which can lead to the recanalization of previously embolized vessels and recurrent hemoptysis over a period of time. To overcome the limitations of gelfoam, some mid‐ or long‐term embolization materials, such as PVA particles and glue, are gradually used as alternatives. As a nonabsorbable embolic material, PVA is widely used to establish a more stable vascular occlusion with available varying particle diameters. Singhal et al. [25] supported that PVA was the preferred agent for embolization. At present, utilization of PVA between 350 and 500 μm in diameter is recommended in clinical practice to improve embolization efficacy and avoid complications. PVA < 300 μm could not only pass through the bronchopulmonary anastomosis, which has a mean diameter of 325 μm in human lung, but also cause very distal embolization occluding the end organs at the precapillary level, which may result in ischemic complications, such as pulmonary infarction and spinal cord injury. However, in the presence of bronchial arterio‐pulmonary shunt, PVA may fail to occlude the culprit vessel; thus, other embolic materials need to be considered.

The use of liquid embolic materials can significantly reduce the rate of recurrence and NBCA has been the most described in the BAE literature [12]. NBCA does not require the thrombotic properties of blood, which is helpful to patients with coagulation dysfunction [22]. The use of NBCA‐based embolic material, which polymerizes quickly after injection, could not only complete the occlusion of the culprit artery but also fill the adjacent potential collateral vessels. By adjusting the proportion of NBCA to iodized oil, controlling the polymerization time, the level of embolization within target vessels and the purpose of controlling bleeding and shortening the procedural and fluoroscopic time were achieved [26]. NBCA is technically challenging to inexperienced operators because it can backflow into the pulmonary or systemic circulation and cause tissue necrosis or other serious complications if inadequately maneuvered. The coil is a permanent embolic material and difficult to recycle once released into the targeted vessel. Then, thrombosis is formed immediately when contacting with blood, to achieve good hemostasis. However, the coil is rarely indicated for BAE, because coil embolization as a proximal occlusion may lead to the rapid formation of distal arterial collaterals. There is limited possibly to repeat embolization of the distal part of the same artery, if the patient has a recurrence of bleeding. Floridi et al. [27] had confirmed that coil used for BAE was associated with a higher recurrence rate and few studies supported that coil treatment was safe and effective for hemoptysis. Now the coil is used primarily to occlude aneurysms and shunts in combination with superselective embolization. Sadidi et al. [28] had proposed that patients with destroyed lungs and embolized arteries wider than 2.0 mm were at higher risk of hemoptysis recurrence in the first year after BAE. However, Figure 4 of this study demonstrated that the vessel responsible for hemoptysis was markedly elongated and dilated. Following the initial embolization with PVA and gelfoam, rapid recanalization occurred; however, hemoptysis was successfully controlled after the second embolization using NCBA, which suggested that embolization strategies should be individualized. According to the anatomical and hemodynamic characteristics of the culprit vessel, operators should choose appropriate embolic materials that they have already used and are comfortable using, keeping in mind the size parameters to mitigate the risk of ischemic complications or shunting into the pulmonary circulation. Sometimes, a combined embolization is necessary. Based on the findings of this research, we preferred PVA and did not use gelfoam alone. However, small quantities of gelfoam in the form of a pledget or thick slurry can be placed after PVA to complete the embolization. When large tortuous bronchial artery with rapid forward flow or arteriovenous shunt was present, NBCA or coil embolization was supplemented.

Kathuria et al. [29] supposed that early recurrence can be attributed to incomplete embolization. Conversely, late recurrence may be caused by the progression of underlying disease, which caused vascular regrowth, dilation, rupture, and bleeding. Frood et al. [30] thought that long‐term survival after BAE was associated with the underlying disease process itself, rather than BAE‐associated adverse events. Therefore, even after successful BAE, active medical treatment measures should be combined based on the etiologies of hemoptysis, including hemostasis, anti‐infection, or antitumor agents therapy. This can prevent the erosion of pulmonary tissues, decrease recurrence of massive hemoptysis, and improve the quality of life. In this study, 30 cases in the control group had embolization technical factors, but they received active medical treatment after the operation, and hemoptysis did not recur. On the contrary, among the 47 recurrent patients in the observation group, 25 cases had the progression of underlying diseases. In this study, multivariate Cox regression analysis showed that the risk factor for late hemoptysis recurrence was the progression of underlying diseases. Therefore, the underlying disease conferring a high risk of hemoptysis recurrence should require more definitive long‐term management. Lu et al. [31] reported that the cystic type of bronchiectasis was a risk factor for recurrence, even in patients receiving BAE, which required long‐term comprehensive management after successful hemostasis. Mahla et al. [32] deemed that although patients with active tuberculosis may present with massive hemoptysis, its response to antituberculous therapy was remarkable.

The common complications associated with BAE include local chest pain, transient dysphagia, and postembolization syndrome, which are usually self‐resolving adverse events. The most severe complication is spinal cord infarction and/or ischemia, leading to transient or persistent paraparesis or paraplegia. This complication is attributed to unintended occlusion of the spinal artery, which originates from the intercostal artery or intercostal–bronchial artery in a small proportion of patients [33]. In this study, there were only common minor complications, which were in agreement with previous studies [34, 35]. No cases of spinal cord infarction were observed.

There were several limitations to this retrospective study, which led to several biases, such as information or selection biases. Small sample size might not be sufficient to identify factors associated with recurrent hemoptysis after BAE. The choice of embolic material was case‐dependent, and patients were not randomly assigned into different modalities for comparison.

## 5 Conclusion

This study preliminarily concludes that the extent of destroyed lung, whether preoperative CTA, technical factors, and the progression of underlying diseases are independent risk factors associated with hemoptysis recurrence following BAE. Adequate preoperative assessment, individualized embolization, and active management of the underlying disease after operation can help reduce the risk of recurrence.

## Author Contributions

Study design: Tianhua Yue. Data acquisition and analysis: Zhengyu Yue, Ling Li. Quality control of data and algorithms: Tianhua Yue. Manuscript editing: Ling Li. Manuscript review: Zhengyu Yue. The author(s) read and approved the final manuscript.

## Funding

This study was supported by Collaborative Innovation Research Project of Jiangsu Medical College (No. 20239220).

## Ethics Statement

The study was approved by the Ethics Committee of Jianhu County People's Hospital (Ethical Approval JY‐LL‐202401‐K023). The Ethics Committee of Jianhu County People's Hospital deemed that patients' consent was not necessary due to the retrospective nature of this study.

## Consent

The authors have nothing to report.

## Conflicts of Interest

The authors declare no conflicts of interest.

## Data Availability

Data generated or analyzed during the study are available from the corresponding author on reasonable request.
